# Temperature-Dependent Gas Transport Behavior in Cross-Linked Liquid Crystalline Polyacrylate Membranes

**DOI:** 10.3390/membranes9080104

**Published:** 2019-08-20

**Authors:** Feras Rabie, Lenka Poláková, Sebastian Fallas, Zdenka Sedlakova, Eva Marand, Stephen M. Martin

**Affiliations:** 1 Department of Chemical Engineering, Virginia Tech, Blacksburg, VA 24061, USA; 2 Institute of Macromolecular Chemistry, Czech Academy of Sciences, Heyrovský Sq. 2, 162 06 Prague, Czech Republic

**Keywords:** liquid crystal polymer, membrane, permeability, nematic, gas separation

## Abstract

Stable, cross-linked, liquid crystalline polymer (LCP) films for membrane separation applications have been fabricated from the mesogenic monomer 11-(4-cyanobiphenyl-4′-yloxy) undecyl methacrylate (CNBPh), non-mesogenic monomer 2-ethylhexyl acrylate (2-EHA), and cross-linker ethylene glycol dimethacrylate (EGDMA) using an in-situ free radical polymerization technique with UV initiation. The phase behavior of the LCP membranes was characterized using differential scanning calorimetry (DSC) and X-ray scattering, and indicated the formation of a nematic liquid crystalline (LC) phase above the glass transition temperature. The single gas transport behavior of CO_2_, CH_4_, propane, and propylene in the cross-linked LCP membranes was investigated for a range of temperatures in the LC mesophase and the isotropic phase. Solubility of the gases was dependent not only on the condensability in the LC mesophase, but also on favorable molecular interactions of penetrant gas molecules exhibiting a charge separation, such as CO_2_ and propylene, with the ordered polar mesogenic side chains of the LCP. Selectivities for various gas pairs generally decreased with increasing temperature and were discontinuous across the nematic–sotropic transition. Sorption behavior of CO_2_ and propylene exhibited a significant change due to a decrease in favorable intermolecular interactions in the disordered isotropic phase. Higher cross-link densities in the membrane generally led to decreased selectivity at low temperatures when the main chain motion was limited by the lack of mesogen mobility in the ordered nematic phase. However, at higher temperatures, increasing the cross-link density increased selectivity as the cross-links acted to limit chain mobility. Mixed gas permeation measurements for propylene and propane showed close agreement with the results of the single gas permeation experiments.

## 1. Introduction

Membrane separation processes are of considerable scientific and economic interest due to their scalability and low energy requirements [1]. They also have the potential to perform separations that are difficult to achieve with other technologies; applications such as desalination, natural gas purification, and paraffin/olefin separation [2,3,4]. Liquid crystalline (LC) materials, which exhibit multiple temperature-dependent mesophases with varying degrees of order, have not received significant scrutiny as potential membrane materials. The ordering of liquid crystalline materials presents a unique opportunity for gas separation processes, which rely on shape and size differences of the penetrants at the atomic level. LC materials offer several options for tuning and controlling mass transport behavior, such as molecular orientation and temperature-dependent phase behavior. The structure and molecular morphology of the membrane material have a direct effect on the gas transport mechanism [5]. Membrane preparation procedures can have significant impacts on membrane performance for a given material [6,7]. In particular, thermal treatment of polymeric films has an effect on gas permeation. Increasing the curing temperature of the polymer films has been shown to increase the packing density of the polymer films, resulting in a decreased gas transport and increased selectivity [8]. For our study, we maintained the curing temperature, pressure, and cooling/heating ramp rate to mitigate any hysteresis effects.

Due to their intrinsic properties, liquid crystalline polymers (LCPs) are attractive in photonic, ferroelectric, and antiferroelectric applications [9,10,11]. LCPs are a distinct class of liquid crystals where the liquid crystal mesogens are attached to a polymer backbone, either by being incorporated in the main chain or attached with a flexible spacer to form a comb-like structure. The flexible spacer decouples motion between the polymer backbone and the LC mesogens, giving the mesogens the ability to form independent phases [12]. In addition, the polymer backbone decreases the macroscopic mobility of the material, which enhances the mechanical stability. The introduction of a crosslinker results in the formation of a network structure that eliminates all macroscopic mobility in the material.

Investigations of the selective pervaporation of volatile organic compounds and the enantioselective properties of cholesteric liquid crystalline membranes have been reported [13,14,15,16]. In addition, there have been several studies of side-chain LCPs focusing on the relationship between polymer microstructure and transport properties [17,18,19,20,21,22]. For example, Kawakami et al. [21] have shown that the overall permeability of a LC polysiloxane with side-chain mesogenic groups was governed by an activated diffusion mechanism, as has been observed in other glassy polymers. In this particular case, the transport properties were dominated by the polysiloxane main chain, which has higher flexibility and free volume. On the other hand, the solubility increased with increasing temperature above the *T_g_*, apparently due to the increased sorption of the penetrant gas in the increasingly disordered liquid crystalline domains. In a series of similar studies, Chen and Hsiue have demonstrated that transport in liquid crystalline polymers does not necessarily follow a biphasic model [23]. In addition, Chen and Hsiue have examined the transport properties of a PDMS side-chain LCP over a large temperature range, spanning the glassy, nematic, and isotropic states of the LCP [18]. Discontinuities in permeation were observed at the glass transition temperature and close to the nematic–isotropic transition. Permeation rates in the isotropic state were very fast and were attributed to the formation of micropores or cracks with convective flow. Sorption of the various penetrants also increased with temperature and the magnitude of this increase was related to the state of order of the mesogenic groups. Chen and Hsiue have also studied cross-linked LC polymers, showing that crosslinking significantly increased the permeability of the side-chain LCPs, most likely because it disrupts the packing order of the mesogenic units [17].

The problem of LCP membrane stability has limited the study and potential industrial applications of these materials. LCPs often have relatively low viscosities in the liquid crystalline state, leading to failure of the membranes. In addition, the mechanism of gas transport through LCP films has not been fully understood. Herein we report on the fabrication and gas transport behavior of cross-linked acrylate-based liquid crystal elastomer membranes, which have a high degree of mechanical stability and can be fabricated into free-standing membranes. To our knowledge, this is the first example of stable, freestanding acrylate LCP gas separation membranes fabricated via in-situ polymerization. Gas permeation behavior has been studied for varying degrees of cross-linking and over a range of temperatures covering the nematic and isotropic phases, providing insight into the mechanism of transport in these LCP materials.

## 2. Materials and Methods

### 2.1. Materials

2-Ethyl hexyl acrylate (2-EHA, b.p. 91 °C/13 mbar, Sigma Aldrich, St. Louis, MO, USA) was distilled with DPPH as a stabilizer under vacuum prior to use. 2-Hydroxy-2-methylpropiophenone (b.p. 110 °C, Sigma Aldrich, 97%), ethylene glycol dimethacrylate (EGDMA, b.p. 98 °C/5 mmHg, Sigma Aldrich, 98%), tetrahydrofuran (THF, b.p. 66 °C, Sigma Aldrich, 99.9%), and ethanol (b.p. 78.4 °C, Decon Labs, 100%) were used as received.

### 2.2. Characterization Methods

Thermal characteristics of the cross-linked acrylate materials were determined using differential scanning calorimetry (DSC). Samples were tested using a Perkin Elmer Diamond DSC (Perkin Elmer, Waltham, MA, USA) with an intercooler 2P unit. Samples were cycled from −50 to 175 °C at a rate of 10 °C/min. Phase transition temperatures were determined using the second heating–cooling cycle.

SAXS and WAXD experiments were performed using a Rigaku S-Max 3000 system (Rigaku, Tokyo, Japan), equipped with a copper rotating anode emitting X-rays with a wavelength of 0.154 nm (Cu Kα). The calibration standard for both SAXS and WAXD was silver behenate. SAXS patterns were obtained using a 2D gas-filled multiwire proportional counting detector, and a Rigaku RAXIA-DI (Rigaku, Tokyo, Japan) was used to read WAXD sample image plates. Exposure times of 2 h were used for all SAXS and WAXD measurements. A Linkam Scientific Instruments temperature controller (Linkam, Tadworth, UK) was used to control sample temperature. A ramp rate of 10 °C/min was used and the sample was allowed to equilibrate for 30 min prior to exposure. The average distance between mesogenic units, d, was calculated using Bragg’s Law.

(1) nλ=2dsinΘ

where λ is the wavelength (0.154 nm) and Θ is the diffraction angle.

Gas permeation runs were performed using a constant volume-variable pressure (single gas) and constant pressure-variable volume (mixed gas) apparatus, which was integrated with a LabView-based computer control and data acquisition system. Data points represent the average of three permeation runs performed for each respective material, penetrant, and temperature. A schematic of the permeation apparatus is depicted in the supporting information.

Permeate volume was determined using a Swagelok 500 mL stainless steel cylinder (Swagelok, Solon, OH, USA). The permeate side was held at approximately 0.2 cm Hg and the cylinder at approximately 1 cm Hg. The two volumes were then equilibrated and the volume of the permeate side was determined using a mass balance. Membrane permeability was determined using the following relation:

(2) $$P=\frac{N_{A}l}{p_{2}-p_{1}}$$

*P* is the permeability, *N_A_* is the gas flux across the membrane, *l* is the membrane thickness, and *p*_2_ and *p*_1_ are the feed and permeation pressures, respectively. Fick’s second law is applied to model the concentration profile for the measurement of solubility and diffusivity by assuming there is no boundary layer resistance at the membrane interface and negligible convective flow through the membrane.

Solving the unsteady state problem results in the following equation for solute diffusivity:

(3) $$D=\frac{l^{2}}{6t_{lag}}$$

For low pressure measurements, the permeability and diffusivity can be related to the solubility via a simple equation [24]:

(4) P=DS

where *D* and *S* are the diffusivity and solubility of a gas in the membrane. Membrane selectivities are defined based on permeability, diffusivity, and solubility as follows: $\alpha_{PAB}=\frac{P_{A}}{P_{B}}$, $\alpha_{DAB}=\frac{D_{A}}{D_{B}}$, and $\alpha_{SAB}=\frac{S_{A}}{S_{B}},$ where the subscripts refer to gas *A* and gas *B*.

Mixed gas permeation runs were performed using a constant pressure-variable volume apparatus. A Shimadzu GC-2014 with a Restek Rt-Alumina PLOT column (Restek, Bellefonte, PA, USA) and an automated 6-port valve were used to separate and measure the concentration of the permeate stream. The permeabilities of propane and propylene under mixed gas conditions were calculated using the following equation:

(5) $$P_{C_{3}H_{i}}=\frac{x_{1,C3Hi}S_{F}l}{x_{s}^{p}A\left(P_{2}x_{2,C3Hi}-P_{1}x_{1,C3Hi}\right)},$$

where *P_C_*_3*Hi*_ is the permeability of propane or propylene. A subscript of 1 denotes the permeate side of the membrane and a subscript of 2 denotes the feed side of the membrane. *P* is the partial pressure of propane and propylene, *x* is the mole fraction of the feed or permeate stream, *S_F_* is the sweep flow rate in the permeate gas (N_2_ or He), *A* is the active separation area, and *l* is the thickness of the membrane.

Permeation is an activated process and can be fitted using an Arrhenius equation [5].

(6) $$P=P_{o}\exp\left(-\frac{E_{A}}{RT}\right).$$

*E_A_* is the apparent activation energy of permeation, *T* is the system temperature, and *R* is the ideal gas constant.

### 2.3. Monomer Synthesis

*Synthesis of the 11-(4-Cyanobiphenyl-4′-yloxy)undecyl methacrylate.* The synthesis of the LC precursor 4′-(11-hydroxy-undecyloxy)-4-biphenylcarbonitrile and the polymerizable LC monomer 11-(4-cyanobiphenyl-4′-yloxy)undecyl methacrylate was performed using a two-step synthesis via a Williamson and esterification reaction and has been described in a previous publication [25].

*Synthesis of 4′-(11-Hydroxy-undecyloxy)-4-biphenylcarbonitrile*. A total of 5.8 g of 4′-hydroxy-4-biphenylcarbonitrile (30 mmol), 11.3 g of 11-bromo-1-undecanol (45 mmol), and 8.0 g of annealed K_2_CO_3_ (60 mmol) were added to 150 mL of dry acetone. The solution was bubbled with nitrogen for several minutes, sealed, and heated to reflux for 72 h. After cooling to a room temperature, solids present in the solution was filtered off. The solvent in the filtrate was removed under reduced pressure. The resulting solid was recrystallized several times in heptane. The chemical structure of the compound was confirmed using NMR (see Appendix A).

*Synthesis of 11-(4-Cyanobiphenyl-4′-yloxy)undecyl methacrylate*. A total of 2.0 g of the synthesized bromide (5.5 mmol) and 830 µL of triethylamine (6 mmol) were dissolved in 20 mL of dry THF. The solution was cooled to −5 °C using ethanol and dry ice. A total of 0.8 mL of freshly distilled methacryloyl chloride (8 mmol) in 5 mL of dry THF was added dropwise to the solution. The solution was stirred at a temperature of 0 to 5 °C for several hours followed by several days at room temperature to ensure that the reaction went to completion. Triethylammonium chloride was filtered off and the solvent in the filtrate was removed under reduced pressure. The resulting solid was recrystallized several times in methanol. The chemical structure of the compound was confirmed using NMR (see Appendix A).

### 2.4. Membrane Fabrication

**Figure 1:**
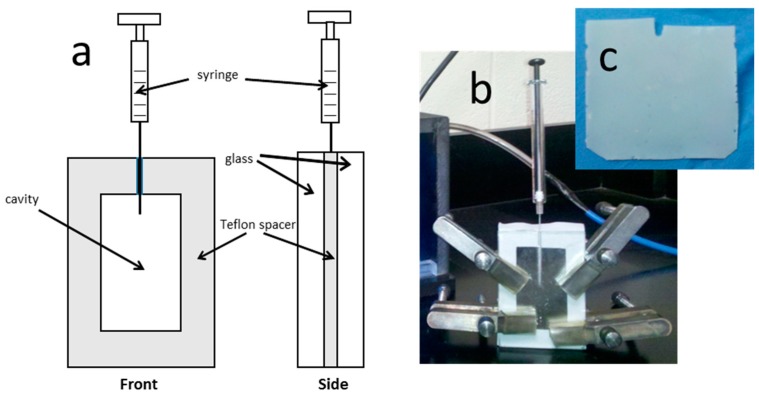
Schematic (**a**) and image (**b**) of the sandwich cell used for membrane fabrication via in-situ free radical polymerization. (**c**) Image of a freestanding membrane following removal from the sandwich cell.

The membrane fabrication apparatus is shown in Figure 1. A dense polytetrafluoroethylene (PTFE) sheet with a rectangular orifice was sandwiched between two glass plates and securely clamped together. A groove in the inner side of one glass plate allowed for the injection of the reaction mixture. The apparatus was designed to minimize solvent loss due to evaporation and inhibition of the reaction caused by the presence of oxygen. The glass plates allowed a UV light source to be used to initiate the polymerization.

**Figure 2:**
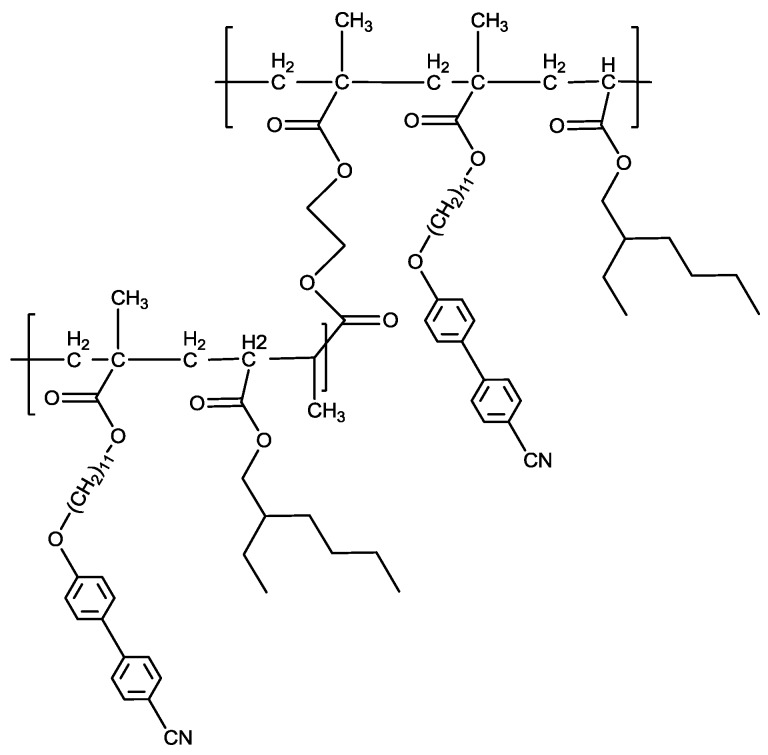
Chemical structure of the cross-linked liquid crystal polymer membrane.

Cross-linked LCP membranes were fabricated using 2.5 or 5.0 mol % crosslinker and 75 mol % LC comonomer (denoted CNBPh75_2.5 and CNBPh75_5.0, respectively) according to the following procedure. A total of 150 mg (0.33 mmol) of 11-(4-cyanobiphenyl-4′-yloxy)undecyl methacrylate, 2.1 μL of EGDMA (0.11 mmol, CNBPh75_2.5), or 4.2 μL of EGDMA (0.22 mmol, CNBPh75_5.0), 22.5 μL (0.11 mmol) 2-EHA, and 1.8 μL (0.12 mmol) Daracure were dissolved in 337.5 mL of THF to achieve a concentration of 1.3 mmol of comonomer per 1 mL of THF. The solution was bubbled with nitrogen for one minute and injected into the membrane reaction chamber. The chamber was blanketed with nitrogen and the injection port was sealed using silicone caulk. The sealed chamber was then exposed to a short-wave UV source (254 nm) for 72 h. Extraction of the membrane and removal of residual unpolymerized components was accomplished with several cycles of solvent swelling and exchange. THF was added dropwise to the membrane followed by immersing the membrane in ethanol for several minutes. The membrane was then placed under vacuum for 48 h to remove residual solvent. An image of the resulting membrane is shown in Figure 1c. The chemical structure of the cross-linked acrylate membrane is depicted in Figure 2. The film thickness was approximately 120 μm. This is the first example of a LC acrylate-based cross-linked film to be produced as a membrane. The membrane was masked using aluminum tape and placed in a permeation cell. To avoid hysteresis and to further minimize any residual solvent effects, the membrane chamber was degassed at 90 °C for 48 h and cooled back to room temperature using a ramp rate of 5 °C/min. All permeation runs were performed from low to high temperature. The membrane and permeation cell were degassed for 5 h between each permeation experiment.

## 3. Results

### 3.1. The Dependence of LC Ordering on Crosslinker and Temperature

**Figure 3:**
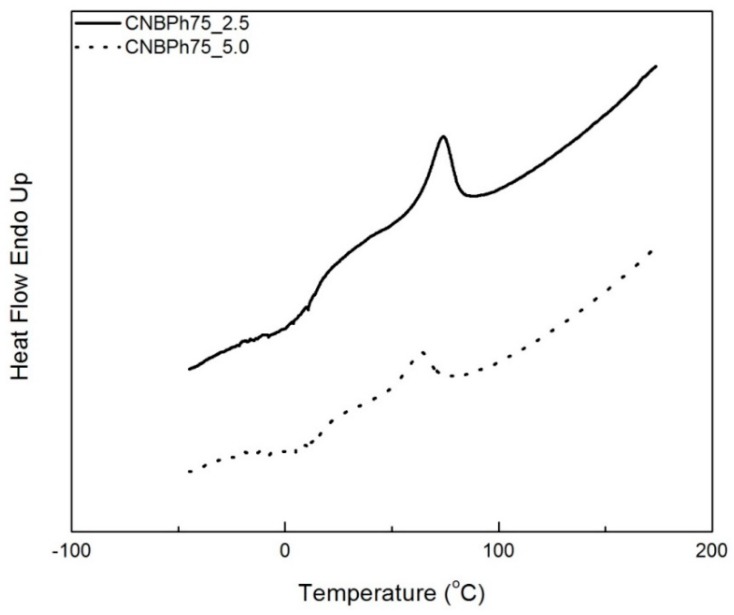
DSC heating curves for the CNBPh75_2.5 and CNBPh75_5.0 membranes.

The phase behavior of the cross-linked CNBPh75_2.5 and CNBPh75_5.0 membranes were characterized using DSC. Figure 3 shows DSC isobars for temperatures from −50 to 150 °C. The glass transition temperature of CNBPh_5.0 (*T_g_* = 17.0 °C) is slightly higher than that of CNBPh75_2.5 (*T_g_* = 12.2 °C) due to the higher degree of cross-linking in the polymer structure. In addition, the temperature of the LC/isotropic phase transition decreased from 73.5 °C for CNBPh75_2.5 to 63.4 °C for CNBPh75_5.0 as the degree of cross-linking increased. Chemical crosslinking imposes constraints on the motion of the chain segments that disrupt the formation of the ordered mesophase [26], resulting in a decrease in the mesophase temperature range. The change in enthalpy of the LC–isotropic transition also decreases with increasing crosslinker content, indicating that the CNBPh75_2.5 samples (Δ*H* = 4.1 J/g) are more ordered than the CNBPh75_5.0 samples (Δ*H* = 2.3 J/g). Results are summarized in Table 1.

**Figure 4:**
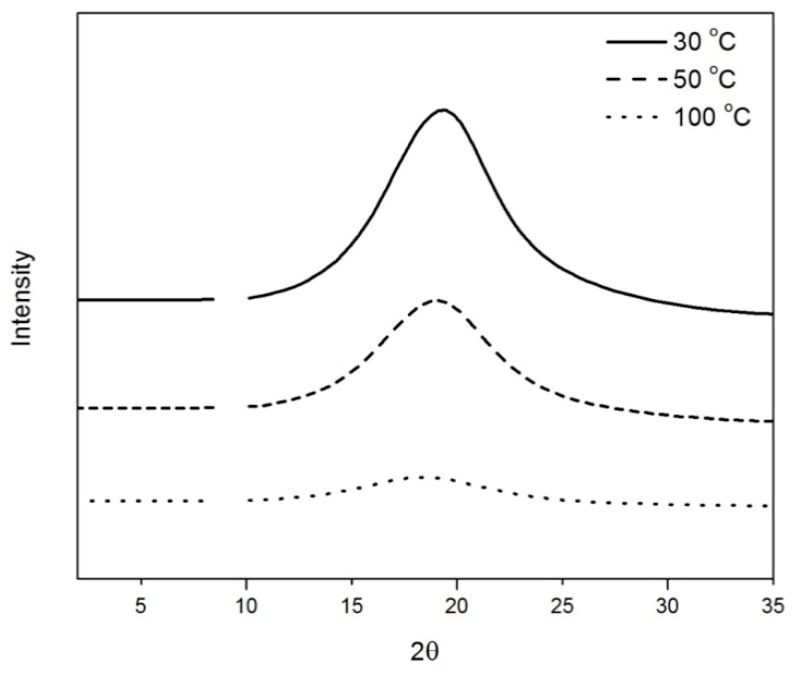
X-ray diffractograms of CNBPh75_2.5 (2.5% crosslinker) at temperatures in the LC mesophase (30 and 50 °C) and isotropic phase (100 °C).

WAXD and SAXS experiments were conducted to characterize the type of LC mesophase present in the films and to determine the effect of temperature on the ordering of crosslinked LC material. Based on the DSC results, an LC mesophase is expected to exist in CNBPh75_2.5 between 12.2 and 73.5 °C and an isotropic phase should occur at higher temperatures. Figure 4 contains SAXS and WAXD data for CNBPh75_2.5 at 30 °C (LC), 50 °C (LC), and 100 °C (isotropic). The absence of a peak in the small angle region and the presence of a broad peak in the wide angle region (2θ = 19.1°) is characteristic of a nematic phase [27]. The corresponding average distance between mesogenic units calculated from the location of the wide angle peak is 4.6 Å. This peak is observed at temperatures of 30 and 50 °C. The WAXD data taken at 100 °C displays only a broad diffuse peak, which is characteristic of an amorphous polymer [28]. The decrease in peak intensities from 30 to 50 °C indicates that the mesogens become less ordered as temperature increases within the mesophase. WAXD measurements on the CNBPh75_5.0 samples also confirmed the presence of the nematic mesophase and the decrease in scattered peak intensity as temperature increases.

### 3.2. Permeability and Diffusivity through LC Crosslinked Membranes

**Figure 5:**
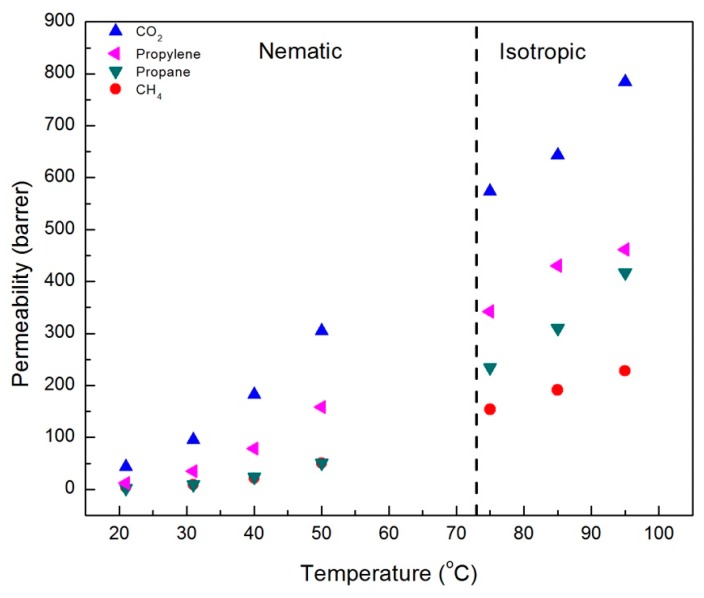
Single gas permeabilities for membrane CNBPh75_2.5 (2.5% crosslinker) plotted as a function of temperature in the nematic and isotropic phases.

**Figure 6:**
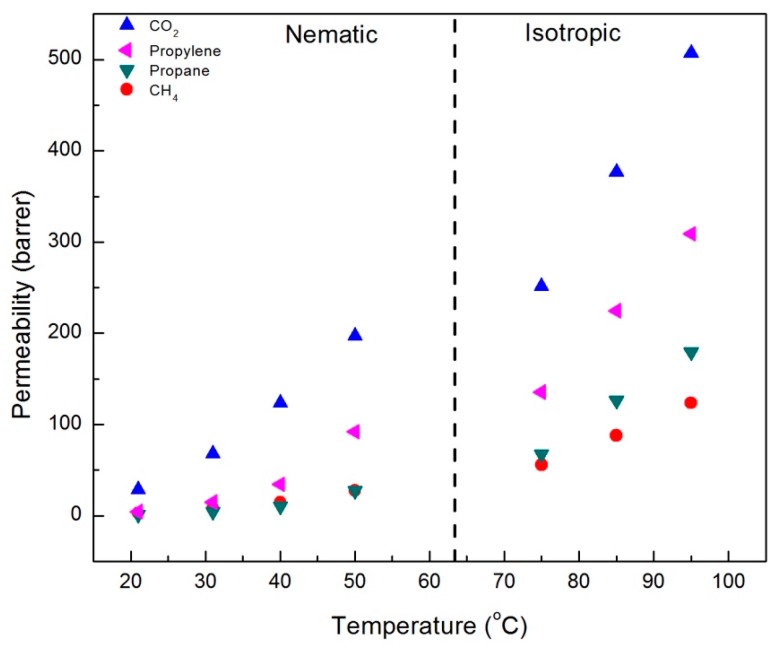
Single gas permeabilities for the CNBPh75_5.0 membrane (5.0% crosslinker) plotted as a function of temperature in the nematic and isotropic phases.

The results of single gas permeability measurements for CO_2_, CH_4_, propane, and propylene in CNBPh75_2.5 and CNBPh75_5.0 membranes are shown in Figure 5 and Figure 6, respectively. The measurements were performed over a range of temperatures both above and below the nematic–isotropic phase transition. As expected, an increase in permeability with increasing temperature is observed for all gases and both membranes. Gas permeabilities increase in the following order; CH_4_, propane, propylene, and CO_2_. A slight discontinuity in the permeation data appeared when transitioning from the nematic phase to the isotropic phase, as demarcated by the dashed line at the phase transition temperature. In addition, increasing the crosslinker content from 2.5 to 5.0 mol% results in a decrease in permeability for all gases studied. Comparison of permeation results in Figure 5 and Figure 6 suggests that the higher crosslinker content reduces the segmental motion of the polymer backbone, leading to a reduction in the diffusivity of the penetrant molecule and a lower permeability [29].

**Figure 7:**
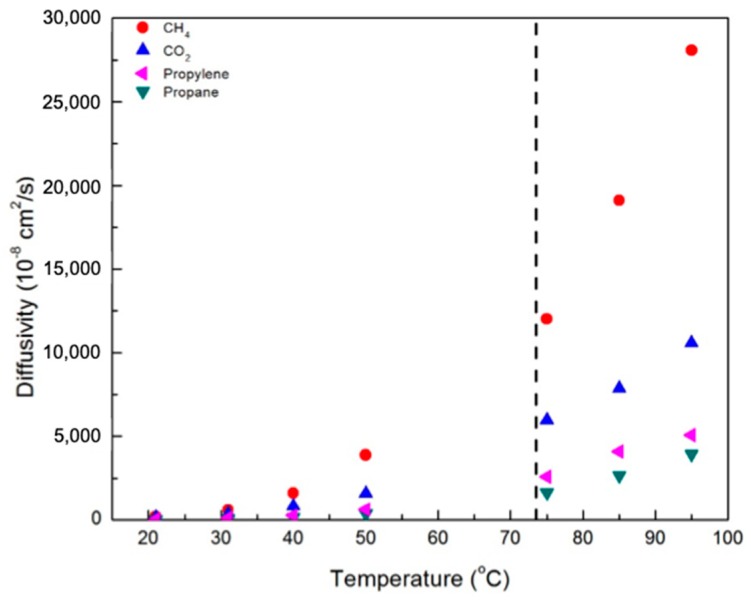
Diffusivity of various gases in the CNBPh75_2.5 membrane (2.5% crosslinker) in the nematic and isotropic phases. The dashed line indicates the nematic–isotropic transition temperature. An expanded view of the nematic region is given in the supporting information.

**Figure 8:**
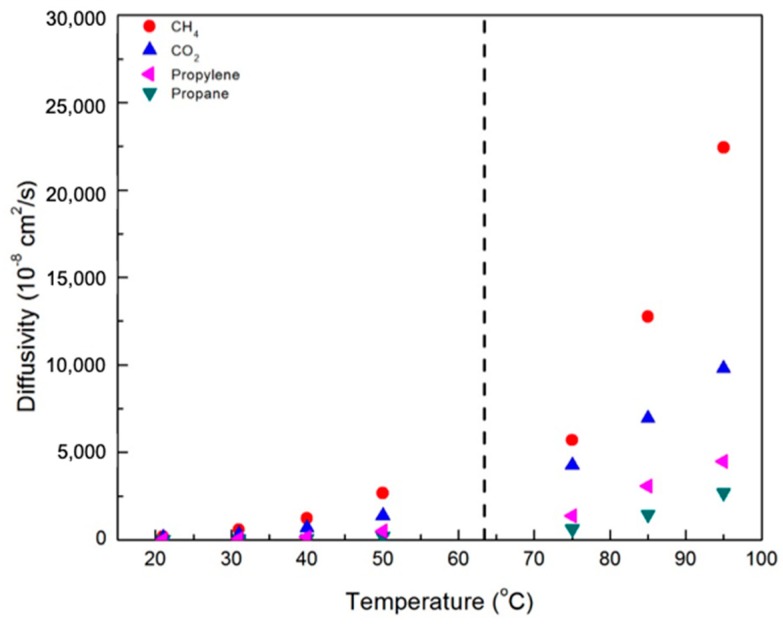
Diffusivity of various gases in the CNBPh75_5.0 membrane (5.0% crosslinker) in the nematic and isotropic phases. The dashed line indicates the nematic–isotropic transition temperature. An expanded view of the nematic region is given in the supporting information.

Figure 7 and Figure 8 contain plots of the diffusivities of gases in the CNBPh75_2.5 and CNBPh75_5.0 membranes, respectively, determined using the time-lag method. Diffusivity increased with temperature and decreased with increasing crosslinker content for all gases studied. This confirms that the increased cross-link density in the CNBPh75_5.0 membrane leads to decreased polymer backbone mobility. Diffusivities for the different gases studied were clearly dependent on the size of the penetrant molecule and decreased with increasing Lennard-Jones gas diameter: CH_4_ (3.76 Å) > CO_2_ (3.94 Å) > propylene (4.68 Å) > propane (5.12 Å). These results are consistent with the kinetic model proposed in the literature, which assumes that the diffusion of molecules occurs as a series of consecutive jumps facilitated by the temporary opening of the polymer matrix as a result of molecular fluctuations [30].

### 3.3. Permeability Selectivity Dependence on Temperature and Crosslink Density

**Figure 9:**
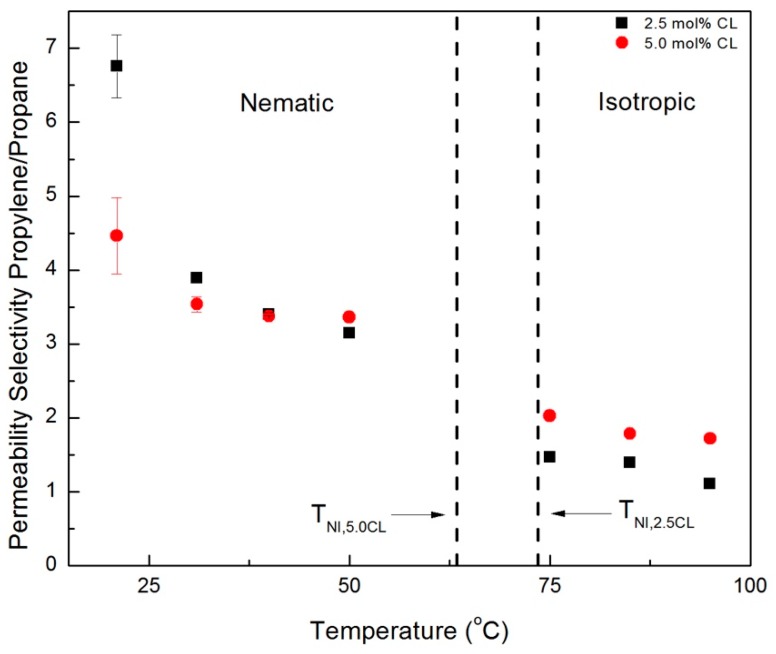
Propylene/propane permeability selectivities in the nematic and isotropic phases for membranes prepared with 2.5% and 5.0% crosslinker (CNBPh75_2.5 and CNBPh75_5.0).

**Figure 10:**
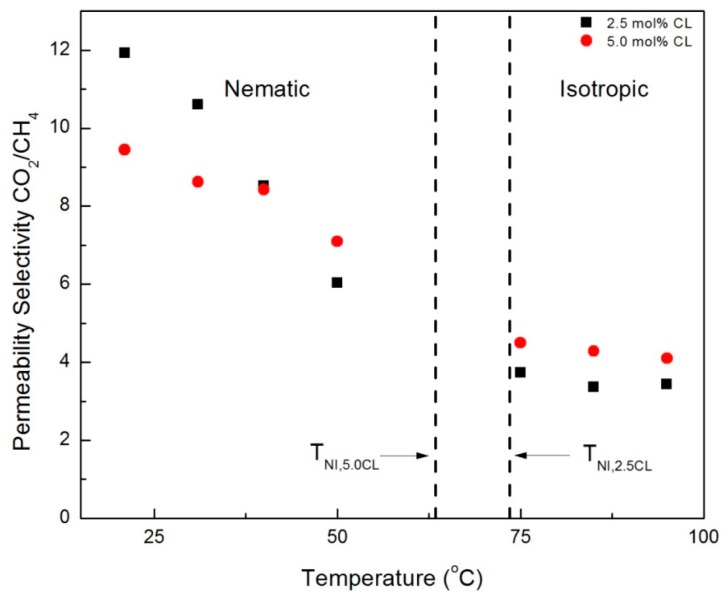
CO_2_/CH_4_ permeability selectivities in the nematic and isotropic phases for membranes prepared with 2.5% and 5.0% crosslinker (CNBPh75_2.5 and CNBPh75_5.0).

Ideal (single gas) permeability selectivity values for the CNBPh75_2.5 and CNBPh75_5.0 membranes were calculated for the gas pairs propylene/propane and CO_2_/CH_4_ and are plotted over a range of temperatures in the nematic and isotropic phases in Figure 9 and Figure 10, respectively. The dashed lines represent the nematic–isotropic transition temperatures for the CNBPh75_2.5 and CNBPh75_5.0 membranes. The value of permeability selectivity decreased with increasing temperature for all gas pairs and for both membranes. The highest observed selectivities were α_propane/propylene_ = 6.7 and α_CO2/CH4_ = 12, which were obtained at a temperature of 21 °C in the CNBPh75_2.5 membrane. In the nematic state, CNBPh75_2.5 had a higher permeability selectivity than CNBPh75_5.0 for all gas pairs. However, the permeability selectivity decreased more rapidly with increasing temperature in the CNBPh75_2.5 membrane so that at a temperature of 35 to 40 °C the CNBPh75_5.0 membrane exhibited higher selectivities. The CNBPh75_5.0 membrane also exhibited higher permeability selectivities for all temperatures and gas pairs in the isotropic state. These results suggest that crosslinking of the LC matrix limits molecular motion at higher temperatures, leading to better overall selectivity.

### 3.4. Diffusion Selectivity Dependence on Temperature and Crosslinker Density

**Figure 11:**
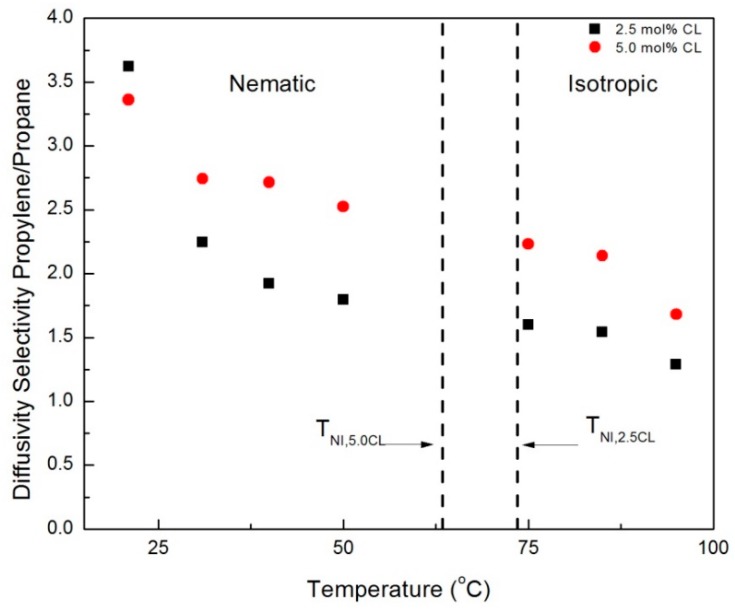
Propylene/propane diffusivity selectivities in the nematic and isotropic phases for membranes prepared with 2.5% and 5.0% crosslinker (CNBPh75_2.5 and CNBPh75_5.0).

**Figure 12:**
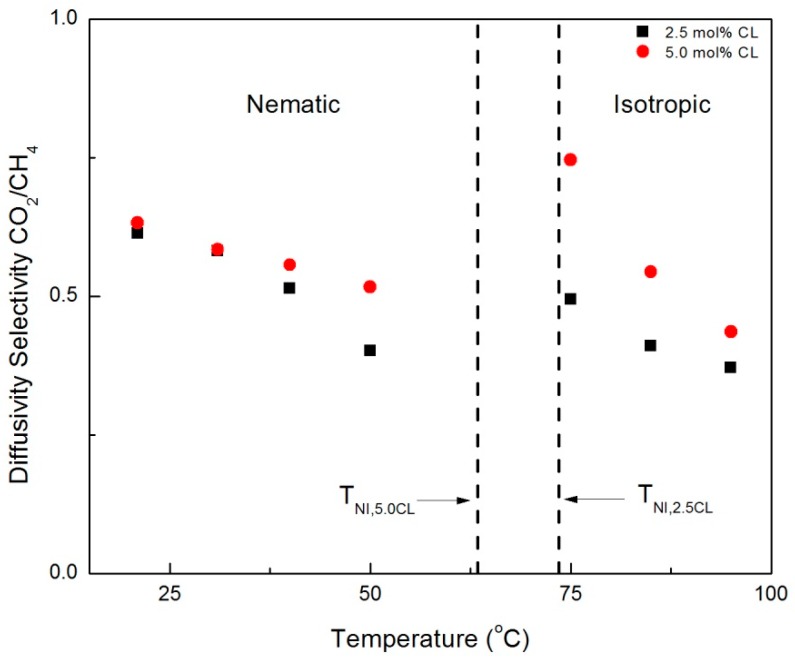
CO_2_/CH_4_ diffusivity selectivities in the nematic and isotropic for membranes prepared with 2.5% and 5.0% crosslinker (CNBPh75_2.5 and CNBPh75_5.0).

The crossover of permeability selectivity between the CNBPh75_2.5 and CNBPh75_5.0 membranes with increasing temperature in the nematic phase may be attributed to differences in the temperature-dependent diffusion selectivity in each membrane. Figure 11 and Figure 12 compare diffusion selectivity in the CNBPh75_2.5 and CNBPh75_5.0 membranes for the gas pairs propylene/propane and CO_2_/CH_4_ over a range of temperatures in the nematic and isotropic phases. Diffusion selectivities for propylene/propane exhibited a dependence on temperature and crosslinker content similar to that observed for permeability selectivities. In the nematic phase, the CNBPh75_2.5 membranes exhibited higher diffusion selectivities at lower temperatures compared to the CNBPh75_5.0 membranes. At higher temperatures, the CNBPh75_5.0 membranes exhibited higher diffusion selectivities. In contrast, the CO_2_/CH_4_ diffusion selectivity was less than one over the whole temperature range, as expected based on the relative Lennard-Jones diameters of the two molecules. The permeability selectivity remains greater than one because of the higher CO_2_ solubility in the membrane. The CO_2_/CH_4_ diffusion selectivity was lower in the CNBPh75_2.5 membrane (i.e., was more selective for CH_4_) than in the CNBPh75_5.0 membranes. In the nematic state, CO_2_/CH_4_ diffusion selectivity in the CNBPh75_2.5 membrane decreased with increasing temperature at a higher rate than CNBPh75_5.0. Increasing the temperature above the isotropic transition resulted in a distinct jump in the CO_2_/CH_4_ diffusion selectivities in both membranes. This jump is not observed for the propylene/propane diffusion selectivities, suggesting that the diffusivity of CO_2_ is more strongly impacted by the ordering in the nematic phase. This could be due to specific interactions between the CO_2_ molecules and the polar cyano group on the mesogenic side chains [31].

WAXD experiments confirmed that the mesogens have higher ordering at lower temperatures in the LC phase compared to higher temperatures in the LC phase. In addition, DSC experiments determined CNBPh75_2.5 has a higher degree of ordering compared to CNBPh75_5.0. This resulted in higher diffusion selectivities at lower temperatures for the CNBPh75_2.5 membrane. This leads to the conclusion that at lower temperatures in the LC phase ordering has a greater effect on polymer mobility and diffusion selectivity than the crosslinking. At higher temperatures in the LC phase, the degree of ordering in both membranes was reduced, resulting in a higher diffusion selectivity for the CNBPh75_5.0 membrane due to the higher crosslink density. Thus, the temperature-driven performance tradeoff between mesogen ordering and polymer backbone mobility controls the diffusivity selectivity of the penetrant gases.

### 3.5. The Effect of LC Phase and Crosslinker Density on Solubility of Gases

**Figure 13:**
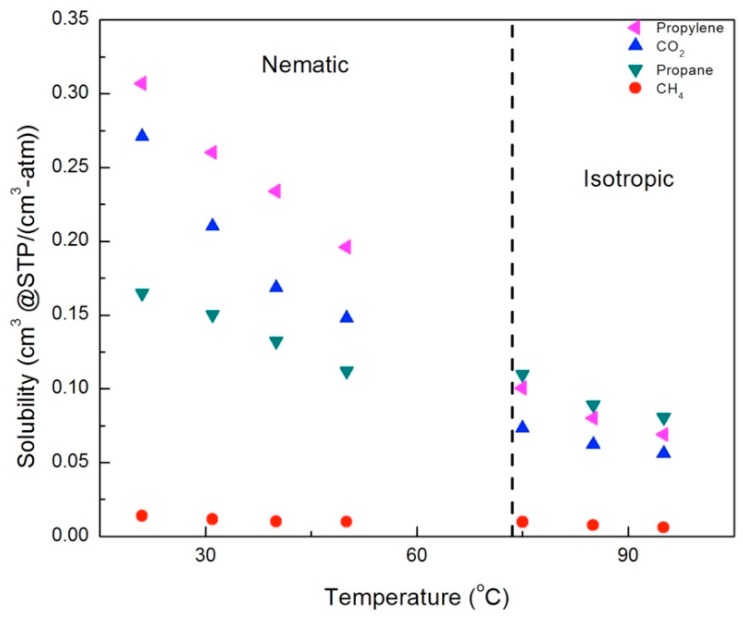
Solubilities for gases in the membrane CNBPh75_2.5 (2.5% crosslinker) in the nematic and isotropic phases.

**Figure 14:**
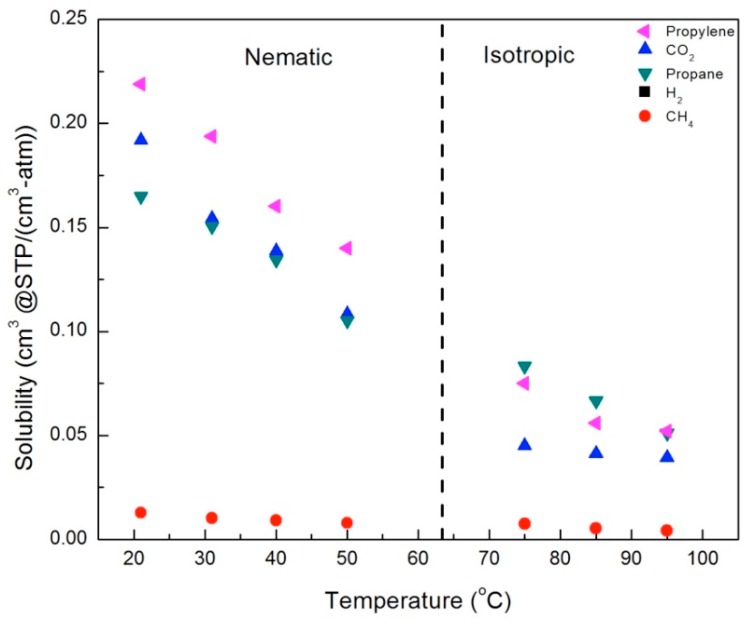
Solubilities for gases in the CNBPh75_5.0 membrane (5.0% crosslinker) in the nematic and isotropic phases.

In order to understand the increase in gas permeability with temperature, we need to separate out the contributions of the gas solubility and diffusion. Calculated solubility values are shown in Figure 13 and Figure 14, for the CNBPh75_2.5 and CNBPh75_5.0 membranes, respectively. The solubility of all penetrants decreases with temperature. This decrease is overcome by the large increase in diffusion, resulting in increasing permeability. Solubilities for propane and CH_4_ were similar in the two membranes; however, the solubilities of propylene and CO_2_ were higher in the CNBPh75_2.5 membrane than in the CNBPh75_5.0 membrane. In general, increasing the crosslink density results in decreased free volume, making sorption of the gas molecules less favorable [32]. In this case, it appears that the effect may be due to the higher crosslink density limiting the potential interactions of the polar mesogenic side chains with the dipole of propylene and the quadrupole of CO_2_.

In general, the solubility decreased with increasing temperature in both the nematic and isotropic phases; however, there were distinct changes in behavior across the nematic–isotropic transition. In the isotropic phase, the relative solubilities of the gases studied ordered from the highest to the lowest solubility are propane > propylene > CO_2_ > CH_4_. In contrast, the ordering of solubilities in the nematic state is propylene > CO_2_ > propane > CH_4_. Thus, propylene and CO_2_, gases with a charge separation, exhibit higher solubilities than propane and CH_4_ in the nematic phase, in both the 2.5% and 5.0% crosslinker membranes. This suggests that increased sorption of CO_2_ and propylene is due to the ordering of the LC mesogens, which enhances the interactions between gases with a charge separation and the mesogenic groups.

### 3.6. Activation Energies of Gas Transport in the Nematic and Isotropic LC Phase

**Figure 15:**
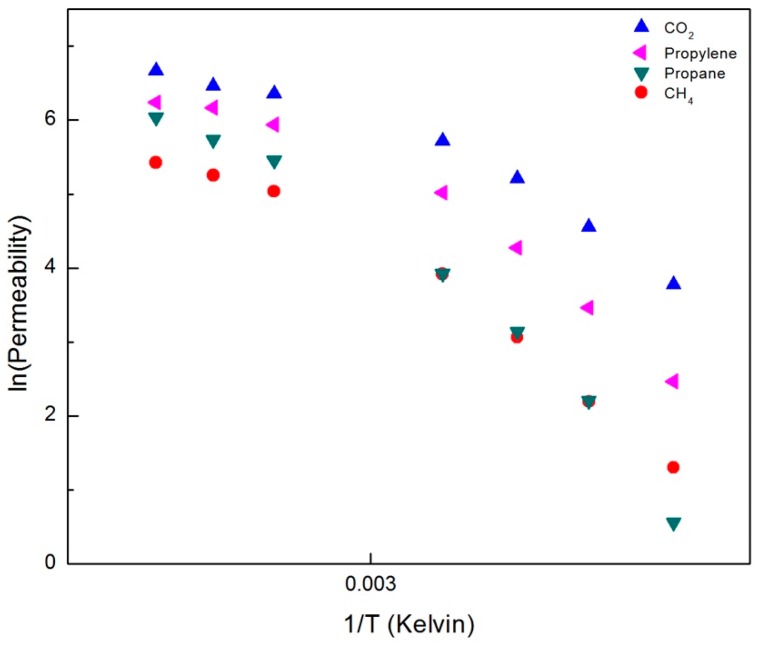
Arrhenius plot of permeabilities for gases in the CNBPh75_2.5 membrane (2.5% crosslinker) in the nematic and isotropic phases.

Permeability is an activated process and follows Arrhenius behavior [5]. Figure 15 is an Arrhenius plot of *ln* (permeability) vs 1/*T* for transport in the CNBPh75_2.5 membrane. Activation energies were obtained from the slopes of the linear fits in both the nematic and isotropic phases and are reported in Table 2. The activation energy for permeation is higher in the nematic phase than the isotropic phase for all gases studied. This is expected, as the ordering in the nematic phase acts as a barrier to transport, and is in agreement with activation energies for transport in small molecule LC materials [16]. The relative activation energies for different gases do not correlate directly with the size of the gas molecules, indicating that interactions between the gases and polymer are probably more important. In addition, the amount of crosslinker had little effect on the activation energies in the nematic state. However, in the isotropic state the activation energies were higher for membrane CNBPh75_5.0, which has a higher degree of crosslinking, which limits the mobility of the polymer chains. These results confirm that the backbone molecular motion controls the primary mode of transport in the disordered phase.

### 3.7. Mixed Gas Permeation of Propylene and Propane

**Figure 16:**
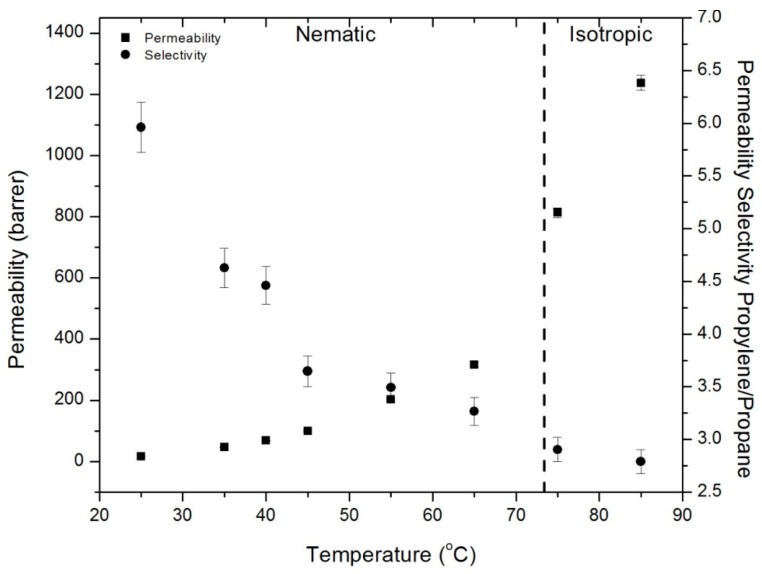
Mixed gas permeability and permeability selectivity for polypropylene and propane in the CNBPh75_2.5 (2.5% crosslinker) membrane in the nematic and isotropic phases.

Mixed gas permeation measurements were performed with a 50:50 propane:propylene gas mixture for CNBPh75_2.5. Figure 16 shows permeability and selectivity measurements for a range of temperatures in the nematic and isotropic states. A propylene/propane permeability selectivity of approximately 6 with a propylene permeability of 17 barrers was obtained at 25 °C. Permeability increased and selectivity decreased with increasing temperature. For all temperatures in the nematic state, mixed-gas propylene permeabilities are in good agreement with single gas runs. The permeability of propane was depressed by the presence of propylene, as evidenced by the higher mixed-gas propylene/propane selectivities compared to the ideal selectivities calculated using single-gas permeation measurements. This behavior is commonly observed in glassy polymers [33]. Table 3 summarizes activation energies for permeation of propane and propylene determined from the mixed-gas permeation experiments. These values are generally slightly lower than those observed in the single gas runs, but follow the same trends.

## 4. Discussion

We developed a membrane fabrication scheme based on the free radical polymerization of a methacrylate cyanobiphenyl monomer with 2-EHA and ethylene glycol dimethacrylate (EGDMA), resulting in the first-reported acrylate-based cross-linked LCP film. Membranes with 2.5 mol % (CNBPh75_2.5) and 5.0 mol % (CNBPh75_5.0) crosslinker were investigated. The extent of ordering in the LCPs was dependent on temperature below the nematic–isotropic transition, and X-ray scattering showed a decrease in molecular ordering in the nematic mesophase with increased temperature. A decrease in the enthalpy of the nematic–isotropic transition was observed at higher crosslinker content, indicating that the mesogens order more efficiently in membranes containing a lower crosslinker content, due to the cross-links limiting the mobility of the mesogenic side chains. An increase in crosslinker content decreased the permeability for all gases tested (CO_2_, CH_4_, propane and propylene) due to a decrease in the diffusivity. Within the LC phase of both membranes, the diffusivity increased with increasing temperature. In addition, the diffusivity of CNBPh75_2.5 (with lower crosslink density) increased at a higher rate with temperature, resulting in lower diffusion selectivity than that observed for CNBPh75_5.0 (with higher crosslink density). At lower temperatures the polymer mobility, and solute diffusivity, is limited due to the ordering of the nematic phases. This effect decreases as temperature rises and at higher temperatures the density of crosslinks dominates the behavior. Trends in the gas solubility indicate that the ordering of the mesogens in the nematic phase enhanced interactions between the polar liquid crystal polymer and penetrant molecules with a charge separation. The solubilities of CO_2_ and propylene were significantly higher in the nematic state than in the isotropic state. Mixed-gas permeation experiments using propylene and propane showed an increase in propylene/propane permeability selectivity compared to that observed for single gas experiments. This result was attributed to the stronger interaction of the propylene with the LC polymer leading to preferential sorption of propylene over propane.

## Figures and Tables

**Table 1:** Phase transitions for CNBPh75_2.5 and CNBPh75_5.0 membranes determined via DSC.

	*T_g_* (°C)	*T_m_* (°C)	Δ*H* (J/g)
**CNBPh75_2.5 (2.5% Crosslinker)**	12.2	73.5	4.1
**CNBPh75_5.0 (5.0% Crosslinker)**	17.0	63.4	2.3

**Table 2:** Activation energies (*E_A_*) for permeation in the nematic mesophase and the isotropic phase for CNBPh75_2.5 (2.5% crosslinker) and CNBPh75_5.0 (5% crosslinker).

Sample	Phase	CO_2_ (KJ/mol K)	Propylene (KJ/mol K)	CH_4_ (KJ/mol K)	Propane (KJ/mol K)
**CNBPh75_2.5**	Isotropic	16.5 (2.9 ^a^)	16.0 (4.6 ^a^)	20.9 (1.40 ^a^)	30.8(1.0 ^a^)
	Nematic	53.3 (2.6 ^a^)	72.3 (3.6 ^a^)	71.8 (1.4 ^a^)	91.2 (9.5 ^a^)
**CNBPh75_5.0**	Isotropic	37.4 (2.7 ^a^)	43.2 (5.0 ^a^)	42.4 (2.8 ^a^)	52.6 (7.6 ^a^)
	Nematic	52.6 (4.1 ^a^)	76.4 (2.2 ^a^)	61.0 (3.2 ^a^)	88.1 (4.8 ^a^)

^a^ Standard deviation of slope -E_A_/R.

**Table 3:** Activation energies for propane and propylene permeation in the nematic mesophase and the isotropic phase for CNBPh75_2.5 (2.5% crosslinker) determined from mixed-gas permeation experiments.

	Propane (KJ/mol K)	Propylene (KJ/mol K)
**Isotropic E_A_**	47.6 (0 ^a^)	43.4 (0 ^a^)
**Nematic E_A_**	71.9 (4.9 ^a^)	65.8 (3.3 ^a^)

^a^ Standard deviation of slope -E_A_/R.

**Figure A1:**
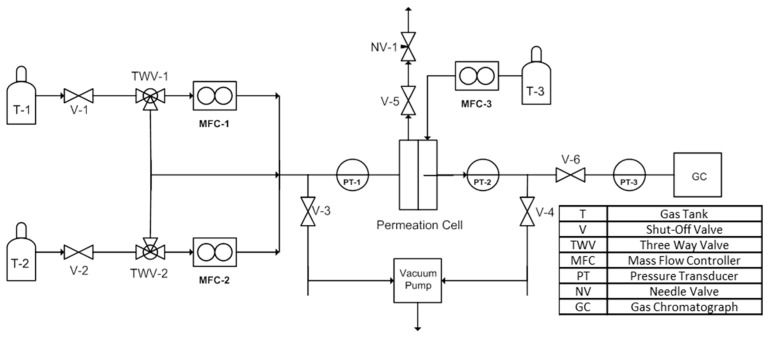
Schematic of the experimental apparatus used for permeation measurements.

**Figure A2:**
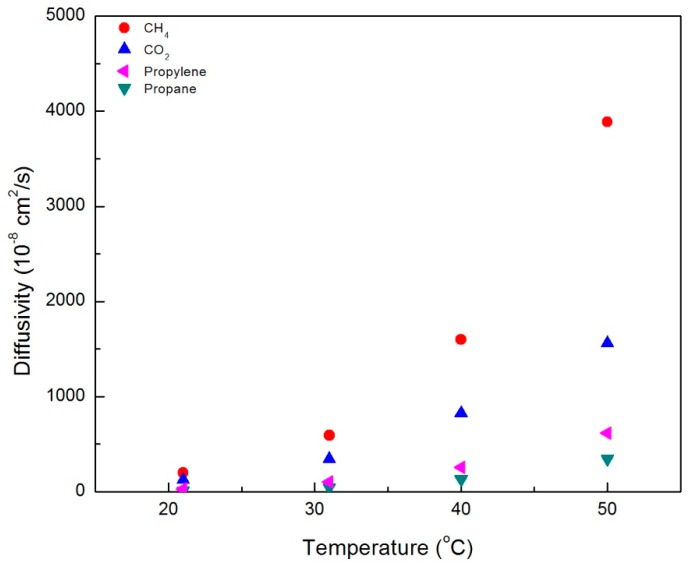
Diffusivity of various gases in the CNBPh75_2.5 membrane (2.5% crosslinker), plotted as a function of temperature in the nematic phases.

**Figure A3:**
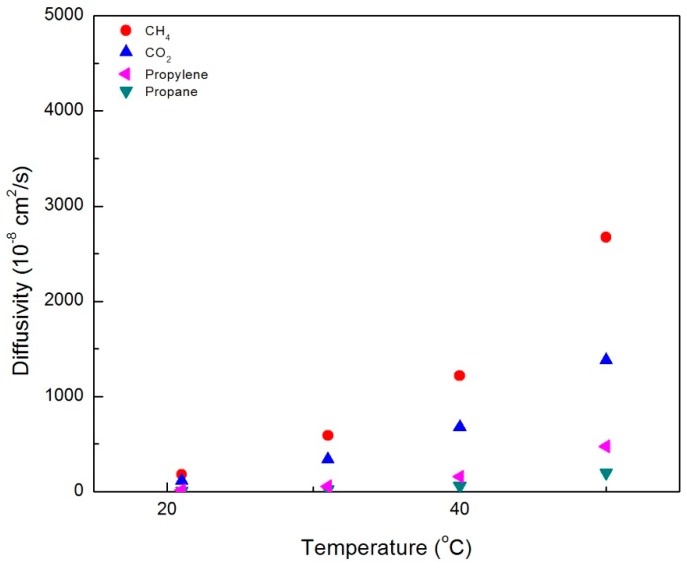
Diffusivity of various gases in the CNBPh75_5.0 membrane (5.0% crosslinker), plotted as a function of temperature in the nematic phases.

## Appendix A

### Monomer Characterization Data

*11-(4-Cyanobiphenyl-4′-yloxy)undecyl methacrylate*: m.p. 98–100 °C, yield—43%. ^1^H NMR data (300 MHz, CDCl_3_): 7.58 (dd, 4H, ortho, meta to –CN), 7.46 (d, 2H, meta to alkoxy), 6.92 (d, 2H, ortho to alkoxy), 3.93 (t, 2H, Ph–O–CH_2_–), 3.58 (q, 2H, –CH_2_–OH), 1.79 (qui, 2H, –O–CH_2_–CH_2_–), 1.19 to 1.56 (m, 16H, aliphatic).

*11-(4-Cyanobiphenyl-4′-yloxy)undecyl methacrylate*: m.p. 75–77 °C, yield—63%. ^1^H NMR data (300 MHz, CDCl_3_): 7.58 (dd, 4H, ortho, meta to –CN), 7.46 (d, 2H, meta to alkoxy), 6.92 (d, 2H, ortho to alkoxy), 5.48 and 6.04 (s + s, 2H, CH_2_ = C(CH_3_)–), 3.93 (t, 2H, Ph–O–CH_2_–), 3.58 (q, 2H, –CH_2_–OH), 1.79 (qui, 2H, –O–CH_2_–CH_2_–), 1.19 to 1.56 (m, 16H, aliphatic).
